# Unusual Case of Simultaneous Presentation of Plasma Cell Myeloma, Chronic Myelogenous Leukemia, and a Jak2 Positive Myeloproliferative Disorder

**DOI:** 10.1155/2014/738428

**Published:** 2014-10-15

**Authors:** J. Maerki, G. Katava, D. Siegel, J. Silberberg, P. K. Bhattacharyya

**Affiliations:** ^1^Rutgers-NJMS, The State University of New Jersey, Newark, NJ 07103, USA; ^2^Hackensack University Medical Center, Hackensack, NJ 07601, USA; ^3^Regional Cancer Care Associates, Freehold, NJ 07728, USA

## Abstract

*Background*. Multiple articles discuss the rare incidence and potential causes of second hematologic disorders arising after treatment of Chronic Myelogenous Leukemia (CML), leading to the theory of imatinib, the current treatment regimen for CML, as a possible trigger for the development of secondary neoplasms. Our case eliminates the possibility of imatinib as the sole cause since our patient received a diagnosis of simultaneous plasma cell myeloma, CML, and a Jak2 mutation positive myeloproliferative disorder (MPD) arising de novo, prior to any treatment. We will further investigate into alternative theories as potential causes for multiple hematopathologic disorders. *Case Report*. There are currently no reported cases with the diagnosis of simultaneous plasma cell myeloma, chronic myelogenous leukemia, and Jak2 positive myeloproliferative disorder. We present a case of a 77-year-old male who was discovered to have these three concurring hematopathologic diagnoses. Our review of the literature includes a look at potential associations linking the three coexisting hematologic entities. *Conclusion*. The mechanism resulting in simultaneous malignancies is most likely multifactorial and potentially includes factors specific to the host, continuous stimulation of the immune system, previous chemotherapy or radiation, a potential common pluripotent stem cell, or, lastly, preexisting myeloma which may increase the susceptibility of additional malignancies.

## 1. Introduction

Plasma cell myeloma, a hematologic neoplasm of lymphoid origin, has an incidence of 4.5 cases per 100,000 person annually in the United States while Chronic Myelogenous Leukemia, a neoplasm of myeloid origin, has an incidence of 1-2 cases per 100,000 person annually worldwide. As coexisting hematologic neoplasms in general are a rare entity, there are only a limited number of case reports with both multiple myeloma and CML [1–13]. Literature review shows case reports of new hematopathologic neoplasms occurring after treatment of an initial hematologic malignancy; this can be a single additional neoplasm or up to three new neoplasms after treatment. Examples of such cases include multiple myeloma after treatment for CML with imatinib [9, 10, 14, 15] and a unique case of three coexisting lymphomas including primary cutaneous marginal zone B cell lymphoma (MZBL), nodal Epstein-Barr virus- (EBV-) associated classic Hodgkin's lymphoma (cHL), and peripheral T cell lymphoma after treatment of nodal T cell lymphoma thirty years prior [16]. The case that we present is exceptionally unique in the fact that it is the only report of three simultaneous hematopathologic neoplasms diagnosed in a patient with no previous exposure to chemotherapy or radiation.

## 2. Case Report

**Figure 1:**
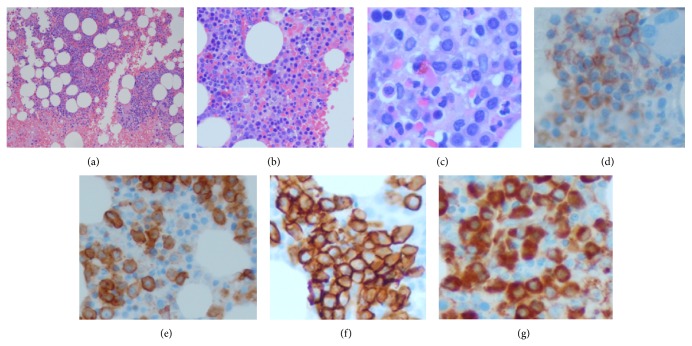
Right posterior iliac crest bone marrow biopsy: (a) H&E 4x, (b) H&E 10x, (c) H&E 20x, (d) CD56 20x, (e) CD117 20x, (f) CD138 20x, and (g) lambda 20x.

The patient, 77-year-old Israeli born male, nonsmoker with no known toxic exposures, was admitted initially for hip discomfort and difficulty in ambulating. Upon initial presentation, the patient's labs were as follows: white blood cell count, 6.2, hemoglobin, 8.7, hematocrit, 25.6, an MCV, 92, platelet count, 242,000, albumin, 3.4, total bilirubin, 2.4, and iron saturation, 15%, with a ferritin of 371. The patient received an extensive work-up at a prior facility with further evaluation by our facility including a cytogenetic analysis at the Mayo Clinic. At the first institution, the patient underwent hip repair, at which time a bone marrow aspiration and bone marrow biopsy were performed. The patient was diagnosed with plasma cell myeloma (Lambda), consisting of approximately 40% of the marrow elements, staining positive for CD138, MUM1, CD117, and CD56. Additionally, the specimen was noted to have multilineage dysplasias including megakaryocytes with hypolobulation organized in occasional clusters and erythroid precursor with megaloblastic changes and irregular nuclear membrane. Lymphocytes were seen scattered in rare benign lymphoid aggregates. The case was reviewed by an outside lab, which was in agreement with the diagnosis of plasma cell myeloma, and the presence of dyserythropoiesis and megakaryocytic variation in size and morphology. Due to the likelihood of an additional underlying hematologic disorder, further testing was completed which included flow cytometric analysis, FISH, cytogenetics, and a complete molecular work-up. Plasma cell myeloma was confirmed with flow cytometry including the presence of hyperploidy with a deletion of 17p. The diagnosis of CML was supported by the t(9;22)/BCR/ABL 1 gene rearrangement (b2a2 type: 4.85%; b3a2 type: 4.85%) producing p210 protein. The presence of a 20q12 deletion was detected by PCR and a JAK-2 V617F mutation was identified which is consistent with a myeloproliferative process. The patient was then seen at our facility in July 2013. The bone marrow biopsy of the right posterior iliac crest showed approximately 60% involvement by plasma cell myeloma and was confirmed by flow cytometry. Immunophenotypic analysis showed positive staining for CD138, CD38, CD56, CD117, and CD27 with cytoplasmic lambda light chain restriction (Figure 1).

The plasma lambda free light chain was elevated to 58.67 mg/L (ref. range: 5.71–26.30 mg/L). The immunofixation results showed two IgA lambda paraprotein peaks and IgA serum levels were elevated at 2144 mg/dL (ref. range: 70–400 mg/dL). Clinically, the plasma cell myeloma was determined as Durie-Salmon stage IIIA. Cytogenetics was sent on a population of the CD138 negative cells to the Mayo Clinic, which showed 20% of the nuclei positive for trisomy 5, trisomy 3/duplication 3q, trisomy 11/duplication 11q, and 17p (TP53) deletion. This was concluded to likely represent the patient's plasma cell dyscrasia. The Mayo Clinic also confirmed the presence of t(9;22)(q34;q11.2) through chromosomal analysis for which RT-PCR verified the fusion of BCR/ABL indicating the presence of early CML. Additionally, the Mayo reported the presence of a JAK2 V617F mutation, providing confirmation for the coexistence of an early myeloproliferative disorder.

After complete work-up at the first institution, the patient received radiation to the right pelvis and femur and was started on a weekly dose of bortezomib. Once the patient was transferred to our facility, and the work-up concluded that the patient had plasma cell myeloma, with an underlying early BCL/ABL positive CML and an underlying Jak2 V617F positive myeloproliferative disorder, the patient continued a weekly treatment of bortezomib and anti-CML therapy was initiated.

## 3. Discussion

There are multiple factors that may attribute to the coexistence of these three hematopathologic diagnoses which include host-specific characteristics, environment, history of previous chemotherapy and radiation, a possible common hematologic stem cell, or the preexistence of plasma cell myeloma. These potential factors and their association to an increase in rate or coexistence of hematopathologic malignancies have been carefully reviewed throughout the literature.

Host-specific factors may include age, gender, race, genetic make-up, and life style choices. The variability in the gene encoding enzymes and repair pathways may predispose a person to increased susceptibility for malignancies [17–19]. Polymorphisms in genes which act in regulation of DNA damage have been found to increase the risk of developing myelodysplastic syndrome and acute myeloid leukemia potentially by proleukemogenic mutations increasing the survival of hematopoietic cells [17, 20]. Life style choices such as tobacco use and alcohol intake are related to multiple primary cancers, and obesity is associated with an increased risk for monoclonal gammopathy of undetermined significance and multiple myeloma [21, 22]. Therefore, patients with a primary malignancy who partake in such behavioral risk factors or who are obese are clearly at higher risk of subsequent malignancies. Additionally, a chronic immunologic stimulation via an infectious, autoimmune, inflammatory, or allergic reaction may give rise to multiple myeloma, acute myelogenous leukemia, and myelodysplastic syndrome [17, 23, 24]. Identifying patients with risk factors would allow the medical team to potentially personalize treatment and surveillance and minimize the risk of secondary malignancies.

Potential environmental influences which may lead to acquired multiple malignancies include radiation exposure and chlorinated solvents but are not limited to these two items. There are some studies linking prior exposure to ionizing radiation to increased risk of developing multiple myeloma, monoclonal gammopathy of undetermined significance, leukemias, and myelodysplastic syndrome [17, 25–29]. Studies have also found a link between exposure to chlorinated solvents and non-Hodgkin lymphoma, leukemia, and multiple myeloma [17, 30, 31]. A thorough history of employment and an individual's living environment may help assess and quantify the risk of developing additional malignancies.

In the literature, there are multiple articles reviewing the association of prior treatment of chemotherapy or radiation and the development of multiple myeloma in the following months particularly imatinib treated CML. Imatinib is the most successful targeted treatment and has transformed the therapy regimen for CML [32]. In a study, imatinib was shown to have an undesirable effect which included the proliferation of multiple myeloma cells through activation of the Erk1/2 mitogen-activated protein kinases [33]. In contrast, another study revealed that imatinib inhibits the proliferation of multiple myeloma cells with arresting cell-cycle progression (Merck and Co., Inc., Whitehouse Station, NJ, http://www.merck.com/). In a fairly recent article, populations of abnormal phenotypes of bone marrow plasma cells have been identified in 21 out of 30 patients with CML who were currently being treated with imatinib. The plasma cells were identified via flow cytometry which included the absence of CD19 in the 21 patients, and 12 of these patients began expressing CD56 [34]. These articles provide support for the effects of imatinib on plasma cells specifically. A conclusion cannot be made at this point as there are conflicting results in published studies and long term effects from imatinib are not well understood due to a relatively short experience with the drug. Additionally, there are only a limited number of cases reported with a posttreatment diagnosis of multiple myeloma in the literature among many patients with CML that have received imatinib. In order to adequately assess the potential relationship between imatinib and multiple myeloma, we must continue to closely monitor the long term effects of imatinib.

Patients with prior tumors that were previously treated with radiation may also be subject to the formation of a secondary malignancy, including CML. Radiation associated CML was investigated by following atomic bomb survivors, women with cervical cancer who were treated with radiation, and patients with ankylosing spondylitis treated with radiation. The results of this study showed a corresponding increase in the diagnosis of CML to the radiation dose [35]. A calculation of 0.0007/Gy has been created to represent the risk of acquiring the translocation (9:22) after exposure to radiation [36]. Because people have been living longer as a result of chemotherapy and radiation treatment, it must be considered that an increase in reported secondary malignancies may be simply due to an increased lifespan. Although the theory of therapy related secondary malignancies may be an accurate etiology for multiple evolving malignant diseases, our case in particular proves that there must also be additional factors that have the potential to give rise to simultaneous hematopathologic malignancies since our patient was not treated prior to diagnosis.

Another potential theory of multiple simultaneous malignancies includes the sharing of a common malignant pluripotent progenitor stem cell which may allow further transformation of both lymphoid and myeloid differentiations. It has been proposed that plasma cell myeloma and CML may evolve from the same hematopoietic stem cell [1, 8, 12]. There have been multiple accounts in which plasma cell neoplasms, including plasma cell leukemia and multiple myeloma, have been seen in coexistence with or arising in the background of CML [37–39]. It is suggested that the simultaneous occurrence of CML and multiple myeloma is analogous to acute lymphoblastic leukemia arising in the blastic phase of CML [5, 6, 8, 12, 15]. This reveals the capability of CML in differentiating into either myeloid lineage or lymphoid lineage. Furthermore, the Philadelphia chromosome (Ph) has been linked to increased cell survival, proliferation, and malignant transformation [1, 40] and has been found in all hematopoietic cell lineages including erythropoietic cells, megakaryocytes, macrophages, and B-lymphocytes [1, 12, 41]. An article highlights 4 cases of multiple myeloma in particular where the Philadelphia chromosome has been identified, providing the potential discovery of a common genetic abnormality among plasma cell neoplasms and CML [42]. In more recent articles, fifteen cases reported to have multiple myeloma and CML were reviewed. Six of these cases revealed the presence of the Philadelphia translocation at the time of diagnosis with multiple myeloma, further supporting a common pluripotent stem cell [1–7]. Moreover, examination of the bone marrow showed a 19% population of myeloma cells and an overall 97% positivity for BCR-ABL fusion signal, indicating that some of the myeloma cells were positive for BCR-ABL rearrangement [1, 2]. On the contrary, 4 of the cases did not show the presence of the Philadelphia translocation; therefore this must not always be necessary for development of simultaneous presentation of both multiple myeloma and CML [1, 8–11]. Another article discusses a case in which multiple myeloma transformed into Ph-negative CML 15 years after the primary diagnosis. In the work-up of the second malignancy, dual-markers for myeloid and plasma antigens were identified on morphologically categorized myeloid cells, indicating the potential presence of a common progenitor cell giving rise to these two entities [43]. Additionally, in the literature, multiple cases of CML are described to have an associated monoclonal immunoglobulin detected. Seven of these eight cases specifically had a monoclonal Ig of lambda isotype. Of these patients, only two were found to have dystrophic cells correlating with the presence of multiple myeloma [44]. This again highlights the overlap between these two diagnoses. There may be a strong indication of multiple hematopathologic malignancies evolving from a common stem cell, although studies have revealed a common progenitor cell is not an essential factor for their coexistence.

Lastly, preexisting plasma cell myeloma may create a more sustainable environment for the formation of secondary malignancies. Plasma cell myeloma is a slow growing malignancy and develops over many years. This in return may lower the effectiveness of the immune system, thereby impeding its ability to destroy newly formed malignant cells. The patient may then present to the clinician with seemingly simultaneous malignancies. Plasma cell myeloma may also initiate the growth of additional leukemias/lymphomas via the numerous potential gene expression profiles and molecular pathways. Studies have revealed the ability of multiple myeloma cells to result in pleiotropic proliferative and antiapoptotic properties [17, 45]. Furthermore, the stimulation of the NF*κ*B signaling pathway has been shown to result in multiple myeloma cell growth, survival, drug resistance, and migration [17, 45]. These properties of multiple myeloma suggest that these changes to the microenvironment of the bone marrow may create sustainability and opportunity for secondary hematopathologic malignancies. A study from Sweden based on 5652 patients with monoclonal gammopathy of uncertain significance (MGUS) revealed an 8-fold increased risk of developing myelodysplastic syndrome and acute myeloid leukemia [46]. Since MGUS is a precursor to multiple myeloma, this authenticates the proposal that molecular pathways created by the progression of multiple myeloma increase the chances of additional malignancies to develop. A better understanding of the molecular profiles and various signaling pathways may allow clinicians to offer targeted therapy and decrease the occurrence of subsequent malignancies.

In our case, the patient presented with all three diagnoses at once prior to treatment and with no known toxic exposure; therefore treatment with imatinib and labor-related or environmental toxins can be given less significance when analyzing our case in particular. Since he presented with all three disorders at once, the consecutive pathophysiology is difficult to identify. However, host-specific factors, development through a common progenitor stem cell, or preexisting plasma cell myeloma leading to the formation of additional hematologic malignancies remain in question. The genetic instability of the host, given his numerous chromosomal abnormalities, may have given rise to an atypical progenitor cell, placing this patient in a more vulnerable position of acquiring multiple disorders. The overlap of plasma cell myeloma and CML described above may also apply to our case in regard to the presence of the Ph chromosome playing a role in the formation of simultaneous plasma cell myeloma and CML. Also, our patient did express a monoclonal Ig, particularly of lambda isotype. This has been seen in multiple CML cases, of which some were shown to have evidence of a secondary diagnosis of multiple myeloma. These two points could potentially indicate the existence of a common stem cell transforming into both plasma cell myeloma and CML. Regarding the relationship of Jak2 and BCR-ABL, it is known that Jak2 is part of the BCR-ABL downstream signaling network and it has been suggested to have a role in increasing the survival of CML Ph positive stem cells. In vitro studies have proposed that BCR-ABL translocation may occur secondary to the existence of Jak2 V617F mutation [47]. Perhaps our patients' Jak2 mutation potentially allowed for increased proliferation and stability of myelogenous leukemic stem cells, giving rise to Ph-positive CML. Lastly, plasma cell neoplasms have been associated with the development of additional hematologic malignancies. The patients multiple disorders were potentially induced via various signaling pathways by his more apparent initial diagnosis of plasma cell myeloma.

In conclusion, further studies need to be completed in order to conclude the exact cause(s) of the rare occurrence of multiple simultaneous malignancies. After close examination of the current literature, it is likely that the coexistence of hematopathologic neoplasms is multifactorial. It is doubtful for this occurrence to be entirely dependent upon one factor since the literature clearly shows variable factors that have likely resulted in secondary malignancies. We also need to consider the diagnosis of these three malignant entities to the possibility of pure chance. The precise mechanism of our extremely rare case remains unknown; hence further investigation and monitoring of potential associated factors are needed.
